# Metronomic topotecan impedes tumor growth of *MYCN*-amplified neuroblastoma cells *in vitro* and *in vivo* by therapy induced senescence

**DOI:** 10.18632/oncotarget.6527

**Published:** 2015-12-09

**Authors:** Sabine Taschner-Mandl, Magdalena Schwarz, Johanna Blaha, Maximilian Kauer, Florian Kromp, Nelli Frank, Fikret Rifatbegovic, Tamara Weiss, Ruth Ladenstein, Martin Hohenegger, Inge M. Ambros, Peter F. Ambros

**Affiliations:** ^1^ CCRI, Chlidren's Cancer Research Institute, Vienna, Austria; ^2^ Department of Pediatrics, Medical University of Vienna, Vienna, Austria; ^3^ Center for Physiology and Pharmacology, Medical University of Vienna, Vienna, Austria

**Keywords:** senescence-associated-secretory-phenotype, metronomic, topotecan, MYCN-amplified neuroblastoma, NFKB1

## Abstract

Poor prognosis and frequent relapses are major challenges for patients with high-risk neuroblastoma (NB), especially when tumors show *MYCN* amplification. High-dose chemotherapy triggers apoptosis, necrosis and senescence, a cellular stress response leading to permanent proliferative arrest and a typical senescence-associated secretome (SASP). SASP components reinforce growth-arrest and act immune-stimulatory, while others are tumor-promoting. We evaluated whether metronomic, i.e. long-term, repetitive low-dose, drug treatment induces senescence *in vitro* and *in vivo*. And importantly, by using the secretome as a discriminator for beneficial versus adverse effects of senescence, drugs with a tumor-inhibiting SASP were identified.

We demonstrate that metronomic application of chemotherapeutic drugs induces therapy-induced senescence, characterized by cell cycle arrest, p21^WAF/CIP1^ up-regulation and DNA double-strand breaks selectively in *MYCN*-amplified NB. Low-dose topotecan (TPT) was identified as an inducer of a favorable SASP while lacking NFKB1/p50 activation. In contrast, Bromo-deoxy-uridine induced senescent NB-cells secret a tumor-promoting SASP in a NFKB1/p50-dependent manner. Importantly, TPT-treated senescent tumor cells act growth-inhibitory in a dose-dependent manner on non-senescent tumor cells and MYCN expression is significantly reduced *in vitro* and *in vivo*. Furthermore, in a mouse xenotransplant-model for *MYCN*-amplified NB metronomic TPT leads to senescence selectively in tumor cells, complete or partial remission, prolonged survival and a favorable SASP.

This new mode-of-action of metronomic TPT treatment, i.e. promoting a tumor-inhibiting type of senescence in MYCN-amplified tumors, is clinically relevant as metronomic regimens are increasingly implemented in therapy protocols of various cancer entities and are considered as a feasible maintenance treatment option with moderate adverse event profiles.

## INTRODUCTION

Patients with metastatic neuroblastoma (NB) frequently suffer from relapse and have a high risk of succumbing to the disease. Neuroblastoma is an embryonic tumor originating from neuronal crest derived neuroblasts and is the most frequent extra-cranial solid tumor in childhood [1, 2]. High-risk neuroblastoma is characterized by tumor cell dissemination into the bone marrow, organ metastasis, amplification of the *MYCN* oncogene (in 40% of cases, personal communication Ladenstein R. and Pötschger U.) and recurrent segmental chromosomal aberrations [3–6]. Despite intensive multi-modal treatment, recent developments in immunotherapy and inclusion of novel targeted drugs in clinical studies, the prognosis of children with high-risk neuroblastoma is still poor, with a median 5-year overall survival between 50 and 70 percent [7–9]. Thus, there is a high interest in new therapeutic strategies that will prevent relapse and improve outcome.

In addition to apoptosis and autophagy, chemotherapeutic agents causing DNA damage result in therapy-induced senescence (TIS), a state of stable proliferative arrest, *in vitro* and *in vivo* [10]. The physiological and pathophysiological role of cellular senescence includes organogenesis during embryonic development [11, 12] and organismal aging [13] as well as removal of damaged cells, as seen upon oncogene-activation in pre-malignant lesions preventing tumor initiation [14]. Even, established tumors gradually regress *in vivo* when senescence is induced by p53 restoration or oncogene inactivation [15–17]. In contrast, senescent stromal cells, i.e. fibroblasts, stimulate the proliferation of premalignant and malignant epithelial cells in culture, and the tumorigenicity of premalignant cells in mouse xenografts [18]. Thus, it is unclear whether TIS – affecting probably both, tumor and stroma – will have tumor-promoting or -inhibiting effects.

Cellular senescence is defined by several features, most importantly, cell cycle arrest accompanied by p21^WAF/CIP1^ and p16^Ink4a^ up-regulation, DNA-damage response (DDR), global chromatin remodeling and epigenetic changes and a characteristic senescence-associated secretory phenotype (SASP) [13, 19–22]. Transcription of SASP factors mainly depend on p38MAPK and nuclear factor kappa B (NFκB) signaling [23, 24]. SASP components of senescent normal cells, oncogene induced senescent premalignant cells and TIS tumor cells comprise autocrine and paracrine factors reinforcing the senescence phenotype, including growth arrest. On the other hand, the SASP includes pro-inflammatory cytokines, growth factors and tissue remodeling enzymes that might act as tumor-promoters [19, 25, 26]. However, SASP-composition differs depending on the genomic background, cell type and senescence trigger. Thus, affecting immune response, apoptotic, angiogenic and mitogenic properties of nearby cells in different ways [20].

TIS has been studied extensively *in vitro*, but *in vivo* data are limited and contradictory. Especially the correlation of TIS with outcome in cancer patients is unclear [27–29]. Currently, several approaches are under investigation to exploit the tumor-inhibiting effects of senescence as cancer therapy [30]. Up until now, studies have focused on TIS induced upon conventional, high-dose cytotoxic drug treatment *in vitro* and in *vivo*. Metronomic, continuous low-dose drug application might also induce TIS. In addition, treatment-related toxic side effects are likely to be reduced under metronomic, low-dose drug schedules. However, current evidence is limited to *in vitro* studies. We have previously shown that long-term low-dose-treatment with hydroxyurea (HU), a ribonuclease reductase inhibitor, induces senescence in primary NB cell lines *in vitro* [31]. This prompted us to screen for additional drugs that induce tumor cell senescence without inducing tumor-promoting properties, such as the unfavorable compartment of the SASP. We further aimed to explore senescence induction by metronomic drug treatment as a new therapeutic strategy for high-risk NB. Therefore, we established an *in vivo* model for low-dose therapy-induced senescence and studied tumor-inhibiting versus-promoting roles of senescent NB-tumor cells and the underlying mechanism *in vitro* and in *vivo*.

## RESULTS

### Long-term, low-dose drug treatment results in cell cycle arrest and senescence in aggressive primary neuroblastoma cell lines

**Figure 1:**
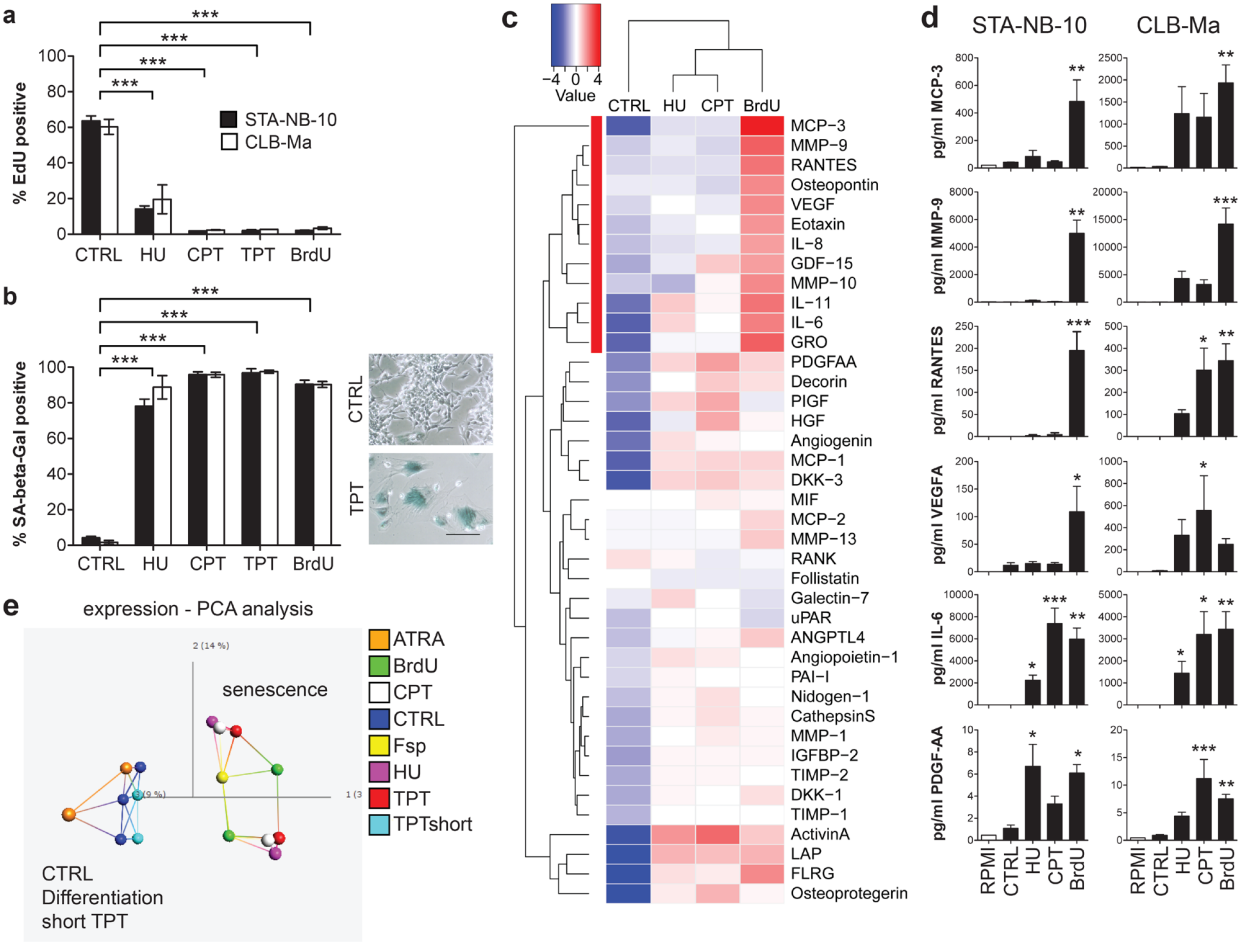
Therapy-induced senescence and differential secretion of tumor-promoting factors in *MYCN*-amplified neuroblastoma cell lines STA-NB-10 or CLB-Ma cells were cultivated in the presence of 150 or 200 μM HU, 3 or 5 nM CPT, resp., 5 nM TPT or 15 μM BrdU for 10 or 3 weeks, respectively. After re-plating **a.** EdU-incorporation and **b.** SA-β-Gal activity was analyzed (*n*= 3). In (b) right panel representative images of TPT-treated and control STA-NB-10 cells after SA-β-Gal staining are depicted. The 100 μm bar refers to control (CTRL) and TPT-treated cells. **c, d.** Cell culture supernatants of CTRL or senescent STA-NB-10 (c, d) or CLB-Ma (d) cells were (c) analyzed by cytokine antibody array for 274 cytokines and growth factors. Secreted proteins with higher than 2-fold difference to CTRL are depicted in the heatmap. Red bar highlights a cluster of unfavorable secreted factors strongly up-regulated in BrdUsen cells. (d) Quantification of MCP-3/CCL7, MMP-9, RANTES, VEGFA and PDGF-AA by ELISA or FACS-based-assays, resp., in supernatants of STA-NB-10 and CLB-Ma treated as indicated. RPMI = complete culture medium without cells. *n* ≥ 3. **e.** PCA blot of microarray gene expression data: senescent NB-cells cluster together and are distinct from differentiatiated cells. Derived from STA-NB-10 and CLB-Ma untreated control (CTRL) cells, differentiation-inducing all-trans retinoic acid (ATRA, 5 μM) treatment for 10 d, spontaneously occurring senescent F-cells (Fsp), short term-TPT for 5 d (TPTshort) and long-term senescence-inducing CPT, TPT, BrdU or HU treatment. Colored lines represent the top 3 levels of proximity acc. to network analysis derived from Qlucore software. Asterisks indicate statistically significant differences. ***p ≤ 0.001; **p ≤ 0.01; *p ≤ 0.05.

In initial dose-finding experiments 12 candidate drugs (Table 1), targeting cell cycle regulation and/or inducing DNA damage, were tested for inhibition of cell proliferation in 6 *MYCN*-amplified (MNA) and 5 non-amplified (nonMNA) NB cell lines (Suppl. Table S1). Two MNA and two nonMNA cell lines showing intermediate drug sensitivities among the cell lines tested were selected for further testing (Table 1 and Suppl. Table S2). Camptothecin (CPT), its derivative topotecan (TPT), distamycin A (DMA), mitoxantrone (MIT), 5-fluorouracil (5-FU), cisplatin (CIS) and the previously published senescence inducers, HU and 5-Bromo-2′-deoxyuridine (BrdU), were tested for induction of senescence in long-term cultures (three weeks) at three different concentrations around the IC50 values (Suppl. Table S2). DMA, MIT, 5-FU and CIS induced cell death, but did not yield senescent cells (Suppl. Table S2). However, HU, CPT, TPT and BrdU treatment resulted – in addition to cell death (Suppl. Figure S1a) - in senescence in both MNA, STA-NB-10 and CLB-Ma, but not in the two nonMNA cell lines (Suppl. Table S2 and Figure 1). Senescent cells had a strongly reduced DNA-synthesis rate, an enlarged, flat morphology, and high senescence-associated-beta-galactosidase (SA-β-Gal) positivity (Figure 1a and 1b). This shows that 4/12 drugs tested, i.e. HU, CPT, TPT and BrdU, lead, in addition to cell death, to senescence in aggressive MNA NB-cell lines, but not in nonMNA cell lines.

**Table 1:** EC50 values after 5d of treatment determined by MTT assay

		EC50 after 5 days treatment [μM]										
		*MYCN*-amplified						*MYCN*-non-amplified				
Substance	target	STA–NB–7	STA–NB–9	STA–NB–10	CLB-Ma	GOTO	Vi856	SK-N-SH	STA-NB-6	CLB-Ga	SK-N-AS	NB-EB
**Camptothecin**	TOP1	n.a.	0.004	0.003	0.004	0.004	0.005	0.007	0.008	0.003	0.023	0.003
**Topotecan**	TOP1	n.a.	0.015	0.006	0.011	0.015	0.011	0.012	0.022	0.008	0.063	0.015
**Mitoxantrone**	TOP2	0.06	n.a.	0.035	0.013	n/a	0.041	0.025	0.263	0.013	0.044	0.053
**Tozasertib**	AURKA	5.4	0.03	0.5	0.08	n/a	0.30	0.2	0.8	0.1	0.4	0.29
**AKI #189406**	AURKA/AURKB/CDK	2.9	0.8	1.0	1.4	n/a	2.1	1.7	1.8	22.7	17.7	0.5
**5-FU**	Nucleoside analog	n.a.	15.4	2.5	4.4	0.8	2.6	n/a	12.9	7.6	4.4	1.2
**Cisplatin**	DNA cross-link	n.a.	1.4	2.7	6.0	2.0	1.3	n/a	4.3	2.1	5.2	n.a.
**AKI #189405**	AURKA/CDK	3.0	1.0	2.7	n/a	n/a	n/a	1.3	n.a.	n.a.	n.a.	n.a.
**DMA**	DNA intercalation	2.4	4.9	9.7	12.6	n/a	5.2	9.2	17.9	8.1	15.8	n.a.
**BrdU**	Nucleoside analog	24.1	17.7	18.5	24.4	n/a	22.5	16.9	558.6	113.7	228.4	n.a.
**Roscovitine**	CDK2	31.7	10.9	56.5	46.8	n/a	23.2	48.6	21.3	28.1	n.a.	n.a.
**Hydroxyurea**	Ribonucleotide reductase	55.7	55.7	167.0	83.0	79.4	115.1	288.6	188.8	117.1	171.1	n.a.

AKI aurora kinase inhibitor; AURKA aurora kinase A; AURKB aurora kinase B; BrdU 5-Bromo-2′-deoxyuridine; CDK cyclin dependent kinase; DMA Distamycin A; 5-FU 5-Fluorouracil; n/a not analyzed; TOP1 topoisomerase I.

### Topotecan induces a favorable SASP independent of NFKB1/p50 activation

Senescent normal cells and neoplastic cells have been reported to produce metastasis- and angiogenesis-promoting factors as part of their SASP [26]. As secretion of these tumor-promoting factors shall be avoided, HU-treated senescent (HUsen), CPTsen and BrdUsen STA-NB-10 cells were analyzed for their secretome. Among the top 40 differentially secreted proteins, a cluster of 12 growth factors and cytokines highly associated with aggressiveness was exclusively secreted at high levels in the BrdUsen cells, but not in the HUsen or CPTsen or the untreated control cells (Figure 1c). Further quantification confirmed that only BrdUsen STA-NB-10 cells secreted the metastasis-related factors MCP-3/CCL7 and MMP-9, the pro-inflammatory protein RANTES and angiogenesis-inducing VEGFA. In contrast, the immune-stimulatory IL-6 and NB-favorable PDGF-AA are secreted by HUsen, CPTsen and BrdUsen cells (Figure 1d). A similar, but slightly distinct, secretion pattern was observed for the CLB-Ma cell line (Figure 1d). Further gene expression profiling confirmed differences in cellular responses as mRNA expression profiles of HUsen TPTsen and CPTsen are similar and clustered more closely together in contrast to BrdUsen, which clustered more distant in both cell lines analyzed (Figure 1e). In contrast to long-term TPT treated cells, short, 5 days, treatment, or all-trans-retinoic acid (ATRA) which mainly induces neuronal-like differentiation in NB, leads only to limited expression changes (Figure 1e). These data illustrate that drug-induced senescent NB-cells undergo similar global expression changes, but secretion of predominantly tumor-promoting SASP factors was confined to BrdUsen cells.

**Figure 2:**
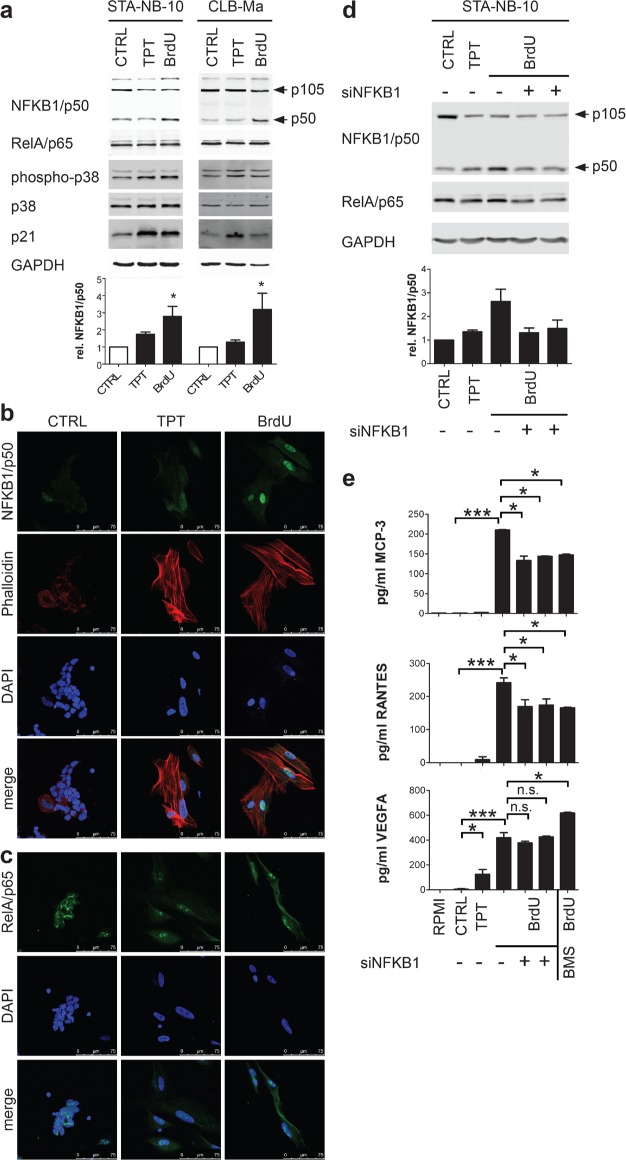
Secretion of unfavorable factors by BrdU-induced senescent NB cells is dependent on NFKB1/p50 **a.** Representative Western blot (upper panel) of CTRL and senescent STA-NB-10 and CLB-Ma treated as indicated; densidometric quantification of NFKB1/p50 relative to GAPDH (*n* = 3; lower panel). **b, c.** Representative confocal image of NFKB1/p50 or RelA/p65, Phalloidin (F-actin) and DAPI IF-staining of STA-NB-10 cells treated as indicated. **d, e.** NFKB1 silencing and pharmacological inhibition in BrdUsen cells: STA-NB-10 cells were treated as indicated and transfected with 2 different siRNAs targeting NFKB1 or non-silencing control siRNA or treated for 24 h with 10 μM BMS345541 (IKKβ inhibitor). Cells and supernatants were harvested 48 h after transfection (medium change 24 h post transfection). (d) Upper panel: representative WB; cells treated as indicated. Lower panel: densidometric analysis (*n* = 3) (e) Quantification of MCP-3/CCL7, RANTES and VEGFA by ELISA (*n* = 3). Asterisks indicate statistically significant differences compared to CTRL. ***p ≤ 0.001; **p ≤ 0.01; *p ≤ 0.05; n.s. not significant.

The transcription of SASP factors was reported to be dependent on the activation of the NFκB and/or p38MAPK pathway [23, 24]. Thus, activation of these pathways was investigated in TPTsen and BrdUsen cells. p38MAPK was phosphorylated in both, TPTsen and BrdUsen cells. However, NFKB1/p50 was up-regulated and showed nuclear localization only in BrdUsen, but not in TPTsen cells (Figure 2a and 2b). The NFKB1/p50 hetero-dimerisation partner RelA/p65 was not increased (Figure 2a and 2c), suggesting that nuclear NFKB1/p50 is required for the transcription of unfavorable SASP factors in BrdUsen cells. SiRNA mediated silencing of NFKB1 reduced secretion of two NFκB target genes, MCP-3 and RANTES, significantly, but did not affect VEGFA ([32, 33] and Figure 2d and 2e). Also pharmacological inhibition of IKKβ, a kinase up-stream of NFKB1/p50, by BMS345541 similarly interfered with MCP-3 and RANTES secretion (Figure 2e). Importantly, these data demonstrate that TPTsen cells produce a more favorable SASP than BrdUsen cells and this is most likely due to a lack of NFKB1/p50 activation.

### Long-term low-dose TPT induces DNA-ds breaks and down-regulation of MYCN

**Figure 3:**
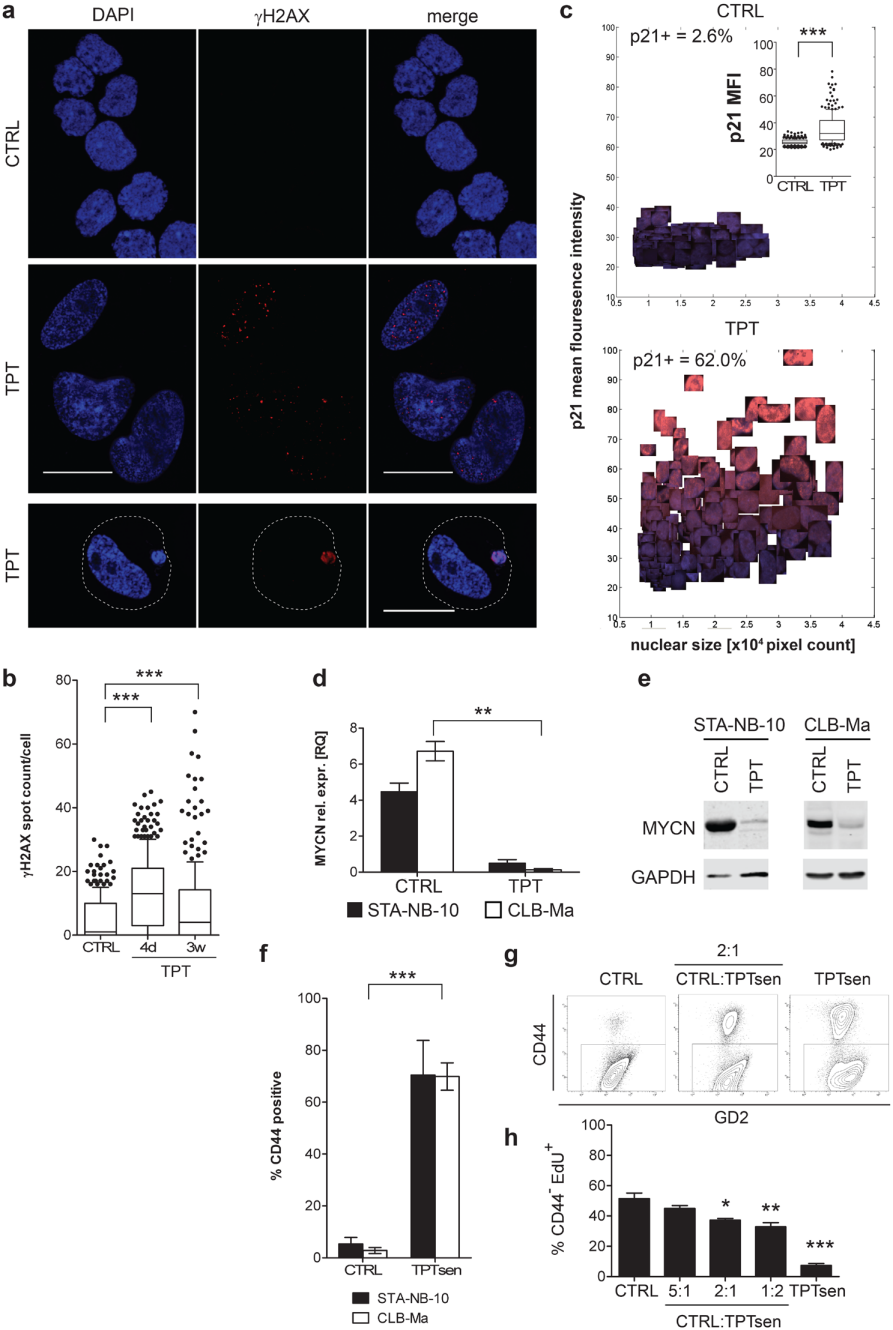
Long-term low-dose TPT-treated senescent NB-cells display senescence-associated markers, down-regulation of MYCN and reduce the proliferation of non-senescent NB cells in co-culture STA-NB-10 or CLB-Ma cells, resp., were cultivated in the absence (CTRL) or presence of 5 nM TPT for 3 weeks. **a.** Analysis of DDB by γH2AX IF staining on cytospin preparations of STA-NB-10 cells treated as indicated (bar: 20 μm) and **b.** quantification by automated imaging. Dashed line in (a) represents the cytoplasmic membrane. **c.** Representative image scatter plots showing clipped nuclei of p21 (red) and DAPI (blue) IF-stained STA-NB-10 cells. Cutoff MFI p21=30; Insert: Box plot showing p21 mean fluorescence intensity of CTRL and TPT-treated STA-NB-10. box plots show mean, box includes 50 percentile, whiskers 10–90 percentile; **d.** qRT-PCR (*n* = 3) and **e.** representative Western blot analysis for MYCN. Bar diagrams depict mean +/− SEM (*n* = 3); **f.** FACS analysis: Mean percentage of CD44+ CTRL and TPT-treated STA-NB-10 (*n* = 3) and CLB-Ma (*n* = 5). **g, h.** After washing, senescent cells were re-plated for co-cultures together with untreated control (CTRL) cells at indicated ratios or as pure cultures. After 7 d of co-culture EdU-incorporation was measured by FACS in the CD44− fraction. (g) Representative FACS plots showing GD2 versus CD44 staining and the gating strategy. (h) Mean % EdU incorporation +/− SEM in the CD44− fraction. ***p ≤ 0.001; **p ≤ 0.01; *p ≤ 0.05.

Next, we examined the mechanism of action of low-dose TPT. High-dose TPT is known to act via topoisomerase I inhibition leading to DNA double strand breaks (DDB) [34]. In order to investigate whether DDBs are present in response to the relatively low doses, i.e. 5 nM TPT applied, nuclear γH2AX foci were analyzed in short-term (4 days) and long-term (3 weeks) TPT treated STA-NB-10 cells. Upon short-term TPT treatment the mean spot count was 3-fold higher as compared to untreated control cells; in long-term TPT treated senescent NB-cells the spot count was 2.2-fold higher, but with a high variance (0 to 70 spots per nucleus) (Figure 3a and 3b). These data indicate that DDB are induced in response to low-dose TPT. Interestingly, some TPTsen cells contain several, small to large micronuclei densely packed with γH2AX positive DNA material (Figure 3a, lower panel), illustrating the elimination of irreparable DNA by packaging in micronuclei. In addition, TPTsen cells up-regulated p21^WAF/CIP1^, but not p16^Ink4a^ in response to DNA damage (Figure 3c and Suppl. Figure S1b) and – in line with previous observations in senescent NB cells [31, 35] - MYCN mRNA and protein expression was abrogated (Figure 3d and 3e).

### TPTsen NB cells inhibit growth of non-senescent NB cells in co-cultures *in vitro*

HU, CPT and TPTsen cells do not secrete a panel of unfavorable, tumor-promoting factors (Figure 1). Thus, we next investigated whether TPTsen cells act tumor-inhibiting or tumor-promoting, i.e. affect proliferation of non-senescent NB-cells *in vitro*. As cell cycle and surface marker stainings cannot be combined with SA-β-Gal staining for FACS analysis, we first confirmed CD44, known to be up-regulated in TIS NB cells, as an adequate surrogate FACS marker to identify senescent NB-cells ([31], Figure 3f and Suppl. Figure S2a). Next, TPTsen cells were co-cultivated with control NB-cells at different ratios for 7 days (Figure 3g and 3h). In TPTsen cells EdU incorporation and Ki-67 were drastically reduced (Figure 3h and Suppl. Figure S2b). In co-cultures, the CD44 negative fraction, containing mainly untreated, non-senescent NB-cells, showed significantly reduced EdU incorporation in a dose-dependent manner – the more TPTsen cells present, the stronger the anti-proliferative effect (Figure 3h). This proves that TPTsen tumor cells do not promote growth of non-senescent neighboring tumor cells but rather reduce their proliferation making TPT a good candidate for further *in vivo* testing.

### Metronomic low-dose topotecan leads to prolonged survival and induces senescence in a dose-dependent manner in a xenotransplantation mouse model

**Figure 4:**
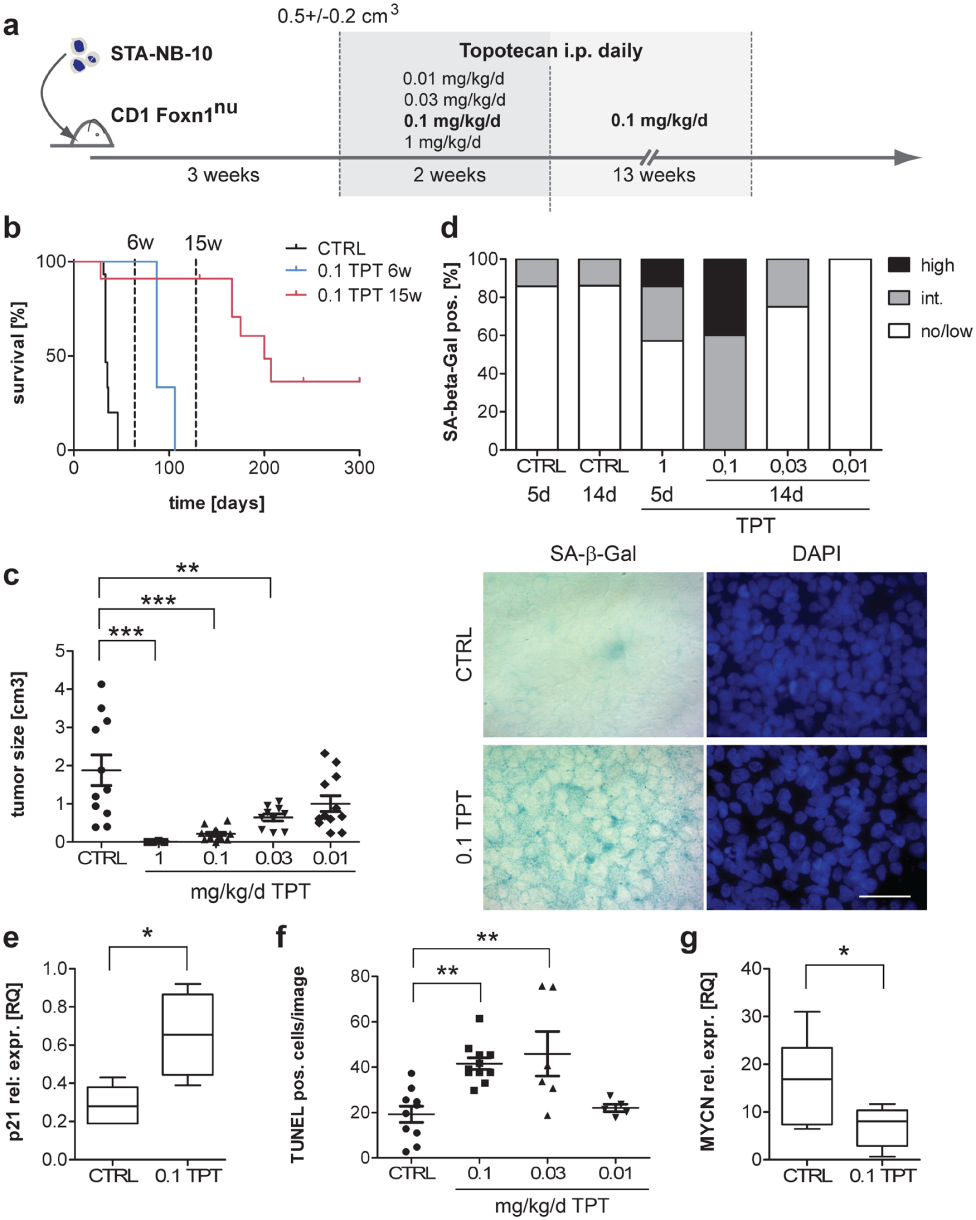
Senescence-induction, reduction of MYCN expression and prolonged survival in a xenograft mouse model for aggressive *MYCN*-amplified NB by continuous low-dose TPT treatment **a.** Experimental setup: STA-NB-10 cells were inoculated s.c. in CD1nude mice and treatment was started at a mean tumor size of 0.53 +/− 0.2 cm^3^ by daily i.p. injection of vector control (CTRL), 1, 0.1, 0.03 or 0.01 mg/kg/d TPT for 2 or 15 weeks, resp. **b.** Kaplan-Meier graph showing survival of control animals (CTRL) (*n* = 15), 6 weeks (*n* = 6) and 15 weeks 0.1 TPT (*n* = 11) treated animals. *p* < 0.0001 (log rank test). **c.** Tumor size and **d.** SA-β-Gal activity after 5 days or 2 weeks of treatment, resp. (d) Upper panel: no/low 0 – 10%; intermediate 10 – 30%; high 30 – 100% of cells SA-β-Gal positive. Lower panel: representative SA-β-Gal and DAPI-staining of tumor touch preparations from 2 weeks treated xenotransplant tumors. **e.** CDKN1A (p21^WAF/CIP1^) qRT-PCR analysis of CTRL (*n* = 5) and 0.1 mg/kg/d TPT treated (*n* = 4) tumors. **f.** TUNEL-staining on cryo-sections of fresh frozen CTRL and TPT-exposed tumors as indicated; line represents mean +/− SEM TUNEL positive cells/image counted on 5 representative areas/tumor. **g.** MYCN qRT-PCR analysis of CTRL (*n* = 6) and 0.1 mg/kg/d TPT treated (*n* = 7) tumors Box plots show mean relative expression values (RQ), box includes 50 percentile, whiskers 10–90 percentile. ***p ≤ 0.001; **p ≤ 0.01; *p ≤ 0.05.

Next, we aimed to establish an *in vivo* model for metronomic drug-induced senescence. TPT has been tested in preclinical studies in xenotransplant tumor models including NB at various doses (0.2 – 2 mg/kg/d), however, TIS was not analyzed and treatment schemes usually consisted of several cycles of treatment including treatment breaks [36, 37]. As our *in vitro* data support TPT as a senescence-inducing drug, when added continuously at relatively low doses (5 nM), for *in vivo* analysis doses ranging from 0.01 to 1 mg/kg/d were applied i.p. daily without treatment break (Figure 4a). According to pharmacodynamics data, 0.1 mg/kg/d yields tissue levels of approximately 5 nM, equivalent to the TPT concentrations used *in vitro* [36]. As, to our knowledge, no syngeneic mouse model exists for NB having a *MYCN* amplification, a xenograft model was established by transplanting the human MNA low passage cell line STA-NB-10 subcutaneously in CD1:*Foxn1^nu/nu^* mice. Treatment was started when the mean longest axis of the xeno-transplanted tumors exceeded 1 cm (Figure 4a).

In control mice, tumors reached the endpoint criteria (*d* ≥ 2 cm) within 14–22 days. When mice were treated for 6 or 15 weeks with 0.1 mg/kg/d TPT, complete (69% at 6 weeks; 91% at 15 weeks) and partial tumor remission, respectively were observed in all animals. 6 weeks 0.1 mg/kg/d treatment led to prolonged survival for another 22–46 days after treatment had been stopped (Figure 4b). If prolonged to 15 weeks, 45% stayed tumor-free for ≥ 300 days (Figure 4b). At week 2 of treatment, tumor size was reduced in a drug-dose dependent manner (Figure 4c). Importantly, in 0.1 mg/kg/d TPT exposed tumors the proportion of SA-β-Gal positive cells and CDKN1A (p21^WAF/CIP1^) mRNA expression was significantly elevated over control, 0.03 or 0.01 mg/kg/d TPT, however only slightly in the 1 mg/kg/d treatment group (Figure 4d and 4e). Notably, stromal cells of murine origin, i.e. endothelial cells, fibroblasts and tumor infiltrating immune cells, did not show altered SA-β-Gal activity in any of the treatment groups (Suppl. Figure S3a). Treatment-related toxic side effects, such as weight loss, splenomegaly or treatment-related deaths have not been observed in the long-term metronomic (0.1 mg/kg/d TPT) treated animals (Suppl. Figure S3b).

Thus, we were able to establish a MNA NB *in vivo* model for metronomic drug-induced senescence, showing that, when applied at low doses *in vivo*, i.e. 0.1 mg/kg/d, TPT triggers senescence in a considerably big proportion of tumor cells, but not in stromal cells and leads to a reduction of tumor size and prolonged survival.

### Continuous low-dose topotecan reduces tumor aggressiveness and does not trigger expression of unfavorable SASP components *in vivo*

**Figure 5:**
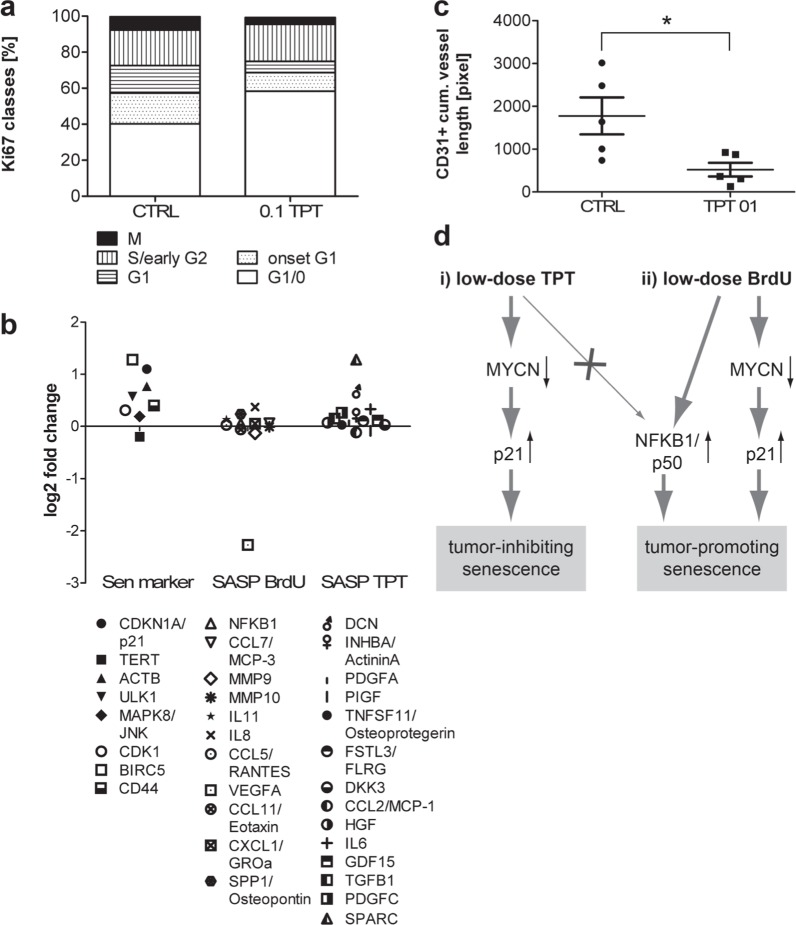
Lack of NFKB1 up-regulation and BrdUsen specific secreted factors in low-dose TPT-exposed tumors *in vivo* **a.** Quantification of Ki-67 IF-staining pattern and corresponding cell cycle state in untreated control (CTRL) and 0.1 mg/kg/d TPT-treated tumors (*n* = 5). **b.** mRNA expression in 0.1 mg/kg/d TPT exposed tumors over control tumors (each *n* = 4). Gene sets: left: senescence related genes (sen marker); middle: factors highly secreted exclusively in BrdUsen *in vitro* (SASP BrdU); right: in TPTsen and BrdUsen *in vitro* (SASP TPT) **c.** CD31 IF-staining on frozen tumor sections. Quantification of CD31^+^ vessel length on 30 random images/tumor (mean cumulative vessel length +/− SEM) in tumors treated as indicated. *p ≤ 0.05. **d.** Model: (i) Metronomic low-dose long-term TPT treatment leads to senescence via p21^WAF/CIP1^ without NFKB1/p50 activation. TPTsen cells down-regulate oncogenic MYCN and act tumor-inhibiting *in vitro* and *in vivo*. (ii) In contrast, BrdU treatment leads to NFKB1/p50 activation and a tumor-promoting SASP.

Previously, apoptosis of tumor cells and reduction of tumor vascularization have been considered as the main mechanisms of action of TPT, leading to tumor shrinkage *in vivo* [38, 39]. Interestingly, apoptosis was elevated to the same extent in response to 0.1 and 0.03 mg/kg/d TPT treatment (Figure 4f), but only in the 0.1 mg/kg/d treatment group senescence was detected in a high proportion of cells (Figure 4d). This indicates that in the 0.1 mg/kg/d dose group senescence probably contributes - in addition to apoptosis - to tumor remission. Next, we evaluated features of aggressiveness in TPT exposed and control tumors, including MYCN expression and proliferative activity. In line with the *in vitro* observation, MYCN expression was diminished by 60% (Figure 4g) and Ki-67 was lower in 0.1 mg/kg/d TPT-exposed tumors (Figure 5a).

Evaluation of SASP expression in our *in vivo* model showed that while several senescence-associated markers were up-regulated in 0.1 mg/kg/d TPT exposed tumors, NFKB1/p50 and SASP-components which were exclusively up-regulated in BrdUsen cells *in vitro*, i.e. unfavorable factors, were not up-regulated upon metronomic TPT *in vivo* (Figure 1c and 5b). In turn, some of the factors typical for TPTsen cells *in vitro*, were up-regulated *in vivo* as well (Figure 5b). Also, metronomic TPT treatment led to a decreased VEGFA expression and reduced tumor vascularization (Figure 5b and 5c). Thus, our *in vivo* data support the notion that metronomic TPT induces senescence in a MNA NB model without up-regulation of NFKB1/p50 and production of a tumor-promoting SASP.

## DISCUSSION

Evidence is accumulating that senescence contributes to cure in various murine and human malignancies [15–17, 27, 28, 40, 41]. We here provide evidence that metronomic, low dose topotecan treatment leads to DNA-damage, p21^WAF/CIP1^ up-regulation, senescence and tumor regression *in vitro* and *in vivo* selectively in MNA NB cells. Topotecan was identified in a small-scale drug screen. By using the SASP as a discriminator for beneficial versus adverse effects of senescence, we successfully screened for drugs that induce a favorable SASP and tumor growth-inhibitory-rather than growth-promoting properties, as demonstrated in co-culture experiments (Figure 5d). The transition to a more favorable phenotype is accompanied by a reduction of MYCN protein, frequently overexpressed in MNA tumors and a marker for bad prognosis. Thus, we propose that metronomic TPT induces a favorable type of senescence in *MYCN*-amplified NB which could be exploited in new therapeutic protocols.

Previous reports have mainly focused on the potential harmful nature of factors secreted by senescent cells. Anti-apoptotic and pro-proliferative effects of senescent fibrosarcoma cell supernatants have been demonstrated in previous co-culture experiments [42]. Here, we identified drugs, HU, CPT and TPT that do not involve secretion of unfavorable SASP factors, such as MMP-9, MCP-3, RANTES and VEGF, but still induce senescence *in vitro* and *in vivo*. The fact that different drugs trigger alternative senescence pathways and cause differences in the secretion profile is in line with earlier observations [43, 44], but this study is the first to exploit this in a drug screen. Regulation of the SASP by NFκB is well established in several cell models [45–47]. We demonstrate that the massive production of tumor-promoting secreted factors in BrdUsen cells is, at least partly, due to the activation of the NFκB-pathway probably involving NFKB1/p50 homodimers, but not RelA/p65 as e.g. in ras-induced senescent fibroblasts [46]. It is tempting to speculate that, based on our *in vitro* data, pharmacological inhibition of the NFκB pathway could reduce some of the tumor-promoting aspects caused by certain drugs, especially when applied at high dose or in a multi-modal setting.

While our data strongly suggest that part of the unfavorable secretome in BrdUsen NB is dependent on NFKB1, VEGFA production was not affected by NFKB1 silencing nor by pharmacological inhibition. In addition, VEGFA expression and vascularization was strongly reduced in metronomic TPT exposed tumors, an effect described earlier for TPT. It has been shown that TPT reduces HIF-1α expression leading to decreased VEGF also in NB and is associated with tumor shrinkage [48–50]. Recently, it has been demonstrated that hypoxia suppresses senescence in normal and cancer cell lines [51]. Thus, DNA-damage induction and alleviation of hypoxic stress by TPT may both contribute to enable and induce senescence.

Metronomic drug application uses continuous low doses of drugs and has been proven effective in phase I and II clinical trials in adult and pediatric solid tumor patients [52–54]. This treatment modality has been assumed to induce tumor cell dormancy and target the tumor microenvironment by reducing the tumor vasculature and by restoring immune surveillance while having little side effects [55], however, the contribution of tumor cell senescence has not been investigated. We here provide evidence that tumor cell senescence might be a major contributor to the tumor-inhibiting effects observed upon low-dose metronomic TPT in *MYCN*-amplified tumors (Figure 5d).

TPTsen cells show down-regulation of MYCN expression *in vitro* and *in vivo*. Extra-chromosomal *MYCN* amplicons have been shown to be subjected to micronucleus-mediated oncogene elimination during TIS and in spontaneously occurring senescent tumor cells in NB [31, 35]. Another mechanism of MYCN down-regulation may involve transcriptional silencing by epigenetic changes taking place during senescence, as exemplified by the formation of repressive heterochromatin at several loci encoding for pro-proliferative genes [57, 58]. In turn, MYCN-down-regulation may itself cause senescence as has been demonstrated for c-myc inactivation [16].

In our *in vitro* cultures we have not observed senescent cells to resume growth upon drug withdrawal, however, a low proportion of cells may cycle very slowly as indicated by low-level DNA-synthesis. It has formerly been shown *in vitro* that replicative senescent fibroblasts having depleted p53 can escape senescence and re-enter cell cycle [59]. It is speculated that upon therapy cancer cells that have accumulated additional mutations relieving cell cycle block might be capable of bypassing or even escaping senescence. Despite these pitfalls, in our study metronomic TPT-induced senescence is associated with tumor remission and prolonged survival. To further reduce relapses, future studies shall combine senescence inducing drugs with drugs targeting metabolic pathways hyperactive in senescence or DNA-repair, e.g. PARP1 inhibitors [60, 61]. Another option may be to boost paracrine activity of infiltrating CD4^+^ effector cells reinforcing senescence or NK-cell mediated clearance of senescent tumor cells [62, 63]. Such combination treatments could enhance the proportion of senescent cells, stabilize cell cycle arrest and/or enable elimination of senescent tumor cells.

Here, we demonstrate in a proof-of-principle study that low dose metronomic TPT does not elicit a tumor-promoting, but rather tumor-inhibiting senescence phenotype due to a lack of NFKB1/p50 activation and leads to tumor remission in an animal model for the *MYCN* amplified, aggressive childhood cancer neuroblastoma. This novel, so far overlooked, effect of metronomic drug treatment is confined to *MYCN*-amplified NB, suggesting that patients with *MYCN*-amplification, could benefit from this treatment modality.

## MATERIALS AND METHODS

### Cell lines, mouse xenotransplantation and drug-treatment

All chemicals and drugs were purchased from Sigma-Aldrich, Austria, unless stated otherwise. NB cell lines (further details see Suppl. Table 1) were maintained in culture as previously described [31]. CLB-Ma was kindly provided by V. Combaret (Centre Leon Berard, France), Vi856 by O. Majdic (Medical University of Vienna, Austria) and SK-N-SH by B. Spengler (Fordham University, USA). GOTO was purchased from the Health Science Research Resources Bank, Japan). Sub-confluent cultures (3–5 × 10^5^ cells/ml) were supplemented with drugs acc. to Suppl. Table 2 (all substances see Table 1; AKI #189405 and #189406 Calbiochem, Austria; Tozasertib LC Laboratories, USA). Treatment with ATRA was carried out for 10 days. Medium containing drugs was replenished 2x/week over a period of 3 or 10 weeks, resp.

For MTT assays cells were seeded at 3 × 10^4^ – 3 × 10^5^/ml in 96 well plates. Drugs were added to triplicate wells in an 8-point dilution series and incubated for 5 days. IC50 values were determined from log-transformed data by nonlinear regression and curve fitting.

Animal studies have been approved by the Medical University of Vienna institutional review board for animal ethics (GZ 66.009/0274-II/3b/2010). 6 to 10 weeks old female CD1*Foxn1*^nu^ mice (Charles River, Germany) were kept under SPF conditions. Mice were inoculated subcutaneously with 10^7^ STA-NB-10 cells in Matrigel (BD Biosciences, Austria) into the right flank. Mice were weighed weekly and tumor size was measured twice a week using a caliper (Tumor volume $V=\frac{\pi }{6}*x*y*z$). Tumors were allowed to grow for 21 days until the mean longest axis ≥ 1 cm (*V* = 0.53 ± 0.2 cm^3^). Topotecan was applied i.p. daily at 0.01, 0.03, 0.1 or 1 mg/kg/d or vector control (0.1% DMSO, PBS) for up to 15 weeks. Mice were sacrificed at day 5 or 14 of treatment or when endpoint criteria (*d* = 2 cm) were met by applying 300 mg Ketamin/30 mg Xylazin i.p. followed by heart puncture. Tumors were measured, weighed and formalin-fixed and paraffin-embedded or cryo-preserved, resp.

### siRNA transfection

siRNA transfection was carried out by lipofectamine-mediated reverse transfection using 2 NFKB1 targeting siRNAs (Silencer Select, s9504 and s9505) and one non-silencing siRNA (Negative control #2, all Life Technologies, Austria) as described [64]. Medium was replenished 24 h post transfection.

### SA-β-Gal staining, immunofluorescence and iFISH

SA-β-Gal staining on cultured cells or tumor touch slides was carried out at pH 5.0 using the Senescence Detection Kit (Biovision, USA) as described [31]. Cultured SA-β-Gal stained cells were imaged using an Axiovert 40C microscope (Zeiss, Austria) and a digital camera (PL-A662; Pixelink, USA). 300 cells/sample were examined for SA-β-Gal positivity by three independent researchers. Tumor touch slides were stained in duplicate, counterstained with DAPI, pseudonymized and allocated into three classes: low (0–10% SA-β-Gal positive of all tumor cells), intermediate (10–30%) and high positivity (30–100%).

Immunofluorescence stainings were performed on 4 μm cryo-sections or cytospin preparations from *in vitro* cultivated cells as described [31]. Briefly, slides were fixed with 4% PFA or methanol-acetone (1:1) and, for detection of nuclear antigens, permeabilized with 0.1% TritonX-100, 0.1% SDS, PBS or 0.3% TritonX-100. Primary antibodies were applied as indicated by the manufacturer (Suppl. Table S3). GD2 antibody (ch14.18) was FITC labelled as described in [31]. Secondary antibodies: anti-m-Cy3, anti-rb-FITC (Dianova) or -AF594, anti-r-AF594 (Life Technologies). For F-actin, 100 nM Phalloidin-TRITC was incubated for 30 min, at RT. TUNEL staining was performed using the DeadEnd Fluorometric TUNEL kit (Promega, Austria) according to the manual.

Confocal images were acquired with a 63x objective at a Leica TCS SP8X laser scanning microscope using the software package LAS AF 4.0.0 (Leica, Germany). Automated image acquisition was done on an Axioplan fluorescence microscope (Zeiss) coupled to a motor-driven stage (Maerzhaeuser, Germany) with a 63x objective using the Metafer Software (V 3.8.6., Metasystems, Germany). Ki-67, γH2AX and p21 were quantified on 100–150 images/sample as described [65, 66]. Quantified cellular properties were nuclear size, nuclear roundness, mean signal intensity, signal background intensity and nuclear spot count. For Ki-67 cells were allocated into six classes according to cell cycle stages based on [67]. Image scatter plots are used to visualize clipped cellular images (combined fluorescence channels), based on iCluster [68]. Images are sorted along the axes using quantified features. CD31 was quantified on 30 random images by Image J (http://rsbweb.nih.gov/ij/download.html) and cumulative vessel lengths were calculated. TUNEL positive cells were quantified by counting positive cells in five areas of comparable cell density by three researchers. *MYCN* interphase-FISH was carried out on cytospin preparations as previously described [31].

### Western blot

Whole cell lysates of cells, SDS-PAGE and Western blotting were done as described [69]. Membranes were probed with primary antibodies (see Suppl. Table S3). Anti-rb or anti-m-IRDye680 or -IRDye800 (Licor, Germany) second step antibodies, resp. were used and detected with the Licor scanner using the Odyssey software (Licor). For densidometric analysis Image Studio Lite V4.0 (Licor) was used.

### mRNA expression analysis

Fresh frozen tumors were homogenized in Trizol (Invitrogen, Austria) by using the MACS Dissociator (Miltenyi, Germany) and total RNA was isolated according to the manufacturer's instructions. 10^4^–10^6^ *in vitro* cultivated cells were used for mRNA-extraction using the miRNEasy kit (Qiagen, Germany). RNA concentration was measured on a Nanodrop ND-1000 (Peqlab, Austria) and RNA integrity was determined by an Experion System (Experion RNA StdSens Analysis Kit, Bio-Rad, Austria) according to the manufacturer's protocol. Following DNAseI digestion and RNA clean-up (RNeasy MinElute Cleanup kit, Qiagen), 200 ng RNA was used for the Primeview Expression Kit (Gene Chip 3′IVT Express Kit, Affymetrix, Austria) and gene expression analysis was performed according to the manufacturer's manual. Further analysis was done in R using Bioconductor packages [70]. CEL files were normalized using the RMA algorithm [71] and the probeset with the highest variance across samples was chosen for further analysis. Microarray data were deposited at the Gene Expression Omnibus (GSE59298). Qlucore Omics Explorer 3.1 (Qlucore, Sweden) was used for PCA analysis and hierarchical clustering.

For quantitative RT-PCR (qRT-PCR), 200 ng of mRNA was used for cDNA synthesis using oligo dT20 primer (VbC Biotech, Austria) and M-MLV reverse transcriptase (Promega) according to the manufacturer's protocol. For qPCR 5 ng cDNA was amplified using the Maxima SYBR Green/ROX qPCR Master Mix (Thermo Scientific, Germany), gene-specific primers (Suppl. Table S4) and the 7500 Fast real-time PCR System (Applied Biosystems, Austria) according to [72]. Normalization and calculation of relative expression values were performed according to [73].

### Cytokine antibody array and ELISA

Cells were washed, seeded at 1 × 10^6^/well for CTRL cells or – to omit confluence – at 5 × 10^5^/well for drug-treated senescent cells in 6 well plates. At day 1, medium was removed and 1.5 ml fresh medium/10^6^ cells was added. After 24 h the supernatant was collected, cellular components were removed by centrifugation and supernatants were snap-frozen. Supernatants were analyzed by G4000 protein array at Raybiotech (USA) for 274 secreted factors. Further analysis was done in R using Bioconductor packages and custom scripts. Background corrected .gpr files were normalized to the internal control and genes with higher than 2-fold difference in the HU, CPT or BrdU-treated senescent NB-cells compared to control were selected for visualization.

Qantitative analysis was carried out by ELISA (MCP-3, Abnova, Taiwan), AlphaLISA (MMP-9, IL-6, RANTES; PerkinElmer, Austria) or Flowcytomix (OPG, VEGFA, PDGF-AA; eBioscience, Austria) according to the manufacturer's instructions. For detection a plate reader (Enspire, PerkinElmer) or the LSRII FACS instrument (BD) was used, respectively.

### Cell cycle analysis and *in vitro* co-culture

*C*ell cultures were supplemented with 10 μM EdU for 14 h, harvested and EdU incorporation was detected by the FACS-based Click-it EdU incorporation kit (Life Technologies) according to the manufacturer's instructions.

Co-culture: 2 × 10^5^ cells/well NB-cells were seeded in 12 well plates as single cultures or were mixed at a ratio of 1:5, 1:2 and 2:1, resp. Medium was replenished every 3 days and cells were passaged, if required. 1 week after culture start, cells were analyzed for cell cycle state by EdU incorporation. Cells were additionally stained with m-anti CD44-PE and anti GD2-FITC and acquired using the BD FACS Fortessa and the Diva software (BD).

### Statistical analysis

If not otherwise stated, Graphpad prism was used for statistical analysis. For parametric analysis ANOVA and Bonferroni posthoc test, for non-parametric analysis the Kruskal-Wallis test was performed. Survival curves were generated by the product limit method of Kaplan and Meier, and compared using the Log-rank test and the Gehan-Wilcoxon test.

## SUPPLEMENTARY FIGURES AND TABLES


