# Supplementary material for: Metronomic topotecan impedes tumor growth of MYCN-amplified neuroblastoma cells in vitro and in vivo by therapy induced senescence
Source: Oncotarget. 2015 Dec 9;7(3):3571–86. doi: 10.18632/oncotarget.6527 (PMC4823128; doi:10.18632/oncotarget.6527)
Supplement: Supplementary file 1 [file oncotarget-07-3571-s001.pdf]

SUPPLEMENTARY DATA

Supplementary Table S1: cell line characteristics

	Cell lines										
	**STA–NB–7**	**STA–NB–9**	**STA–NB–10**	**CLB-Ma**	**GOTO**	**Vi-856**	**SK-N-SH**	**STA-NB-6**	**CLB-Ga**	**SK-N-AS**	**NB-EB**
INSS stage	3	4	3	4	4	4	4	3	4	4	4
Patient outcome	CR	CR	DOD	n.a.	DOD	DOD	DOD	CR	n.a.	n.a.	n.a.
MYCN amplification	Yesdmin	Yesdmin	Yesdmin	Yesdmin	YesHSR	Yesdmin	No	No	No	No	No
17q gain	Yes	Yes	Yes	Yes	Yes	Yes	Yes	Yes	Yes	No	No
1p loss	Yes	Yes	Yes	Yes	Yes	Yes	No	Yes	Yes	yes	yes
ref		[1, 2]		[3]	[4]	[2, 5, 6]	[7]	[1, 2]	[3]	[8].	[9]

CR clinical remission; DOD dead of disease; dmin double minutes; HSR homogenously staining regions; INSS international neuroblastoma staging system; n.a. not known.

Supplementary Table S2: Optimal drug concentration for *in vitro* senescence induction according to phenotype and SA-β-Gal activity

		Concentration optimal for *in vitro* senescence induction			
		MYCN-amplified		*MYCN*-non amplified	
**Substance**	**Concentrations tested**	**STA–NB–10**	**CLB-Ma**	**SK-N-SH**	**STA-NB-6**
Camptothecin	1, 3, 5 nM	3 nM	5 nM	no sen	no sen
Topotecan	1, 3, 5 nM	5 nM	5 nM	no sen	no sen
BrdU	15, 20, 25 µM	15 µM	15 µM	no sen	no sen
Hydroxyurea[1)]	100, 150, 200 µM	150 µM	200 µM	no sen	no sen
DMA	5, 10, 15 µM	no sen	no sen	no sen	no sen
Mitoxantrone	10, 20, 40 nM	no sen	no sen	no sen	no sen
5-FU	2, 3, 5 µM	no sen	no sen	no sen	no sen
Cisplatin	2, 3, 6 µM	no sen	no sen	no sen	no sen

no sen, no senescent cells present after 3 weeks cultivation;
[1)]10 weeks treatment.

Supplementary Table S3: Antibodies used in this study

Antigen	Clone	Species	Conjugated	Dilution	Company	Application
Phospho-(Ser139)-H2AX	JBW301	mouse	-	1:500	Millipore, Austria	IF
Ki-67	MM1	mouse	-	1:100	Novocastra, Austria	IF
CD31	S231	rat	-	1:100	Dianova, Germany	IF
$p21^{WAF/CIP1}$	SX118	mouse	-	1:50	DAKO, Austria	IF
$p16^{Ink4a}$	F-12	mouse	-	1:500	Santa Cruz, USA	IF
NFKB1/p50	H-115	rabbit	-	1:50/1:200	Santa Cruz, USA	IF/WB
p65/RelA	C-20	rabbit	-	1:50/1:200	Santa Cruz, USA	IF/WB
CD44	BMS113	mouse	-	1:50	Bender MedSystems, Austria	IF
GD2	ch14.18	chimeric	FITC	1:100/1:200	Polymun, Austria	IF/FACS
MYCN	NCM II 100	mouse	-	1:50	Abcam, Austria	WB
GAPDH	6C5	mouse	-	1:1000	Santa Cruz, USA	WB
phospho-(Thr180/Tyr182)-p38MAPK	9215S	rabbit	-	1:100	Cell Signaling, Austria	WB
p38MAPK	L53F8	mouse	-	1:100	Cell Signaling, Austria	WB
$p21^{WAF/CIP1}$	OP-64	mouse	-	1:200	Calbiochem, Austria	WB
CD44		mouse	PE	1:100	Becton Dickinson, Austria	FACS

Supplementary Table S4: Primer used in this study

Gene	Forward primer sequence	Reverse primer sequence	ref
HPRT1	5′-TGACACTGGCAAAACAATGCA-3′	5′-GGTCCTTTTCACCAGCAAGCT-3′	[10]
SDHA	5′-TGGGAACAAGAGGGCATCTG-3′	5′-CCACCACTGCATCAAATTCATG-3′	
UBC	5′-ATTTGGGTCGCGGTTCTTG-3′	5′-TGCCTTGACATTCTCGATGGT-3′	
CDKN1A	5′-GCAGACCAGCATGACAGATTT-3′	5′-ACACACAAACTGAGACTAAGGCA-3′	this study
MYCN	5′-CCGGGCATGATCTGCAA-3′	5′-CCGCCGAAGTAGAAGTCATCTT-3′	[11]

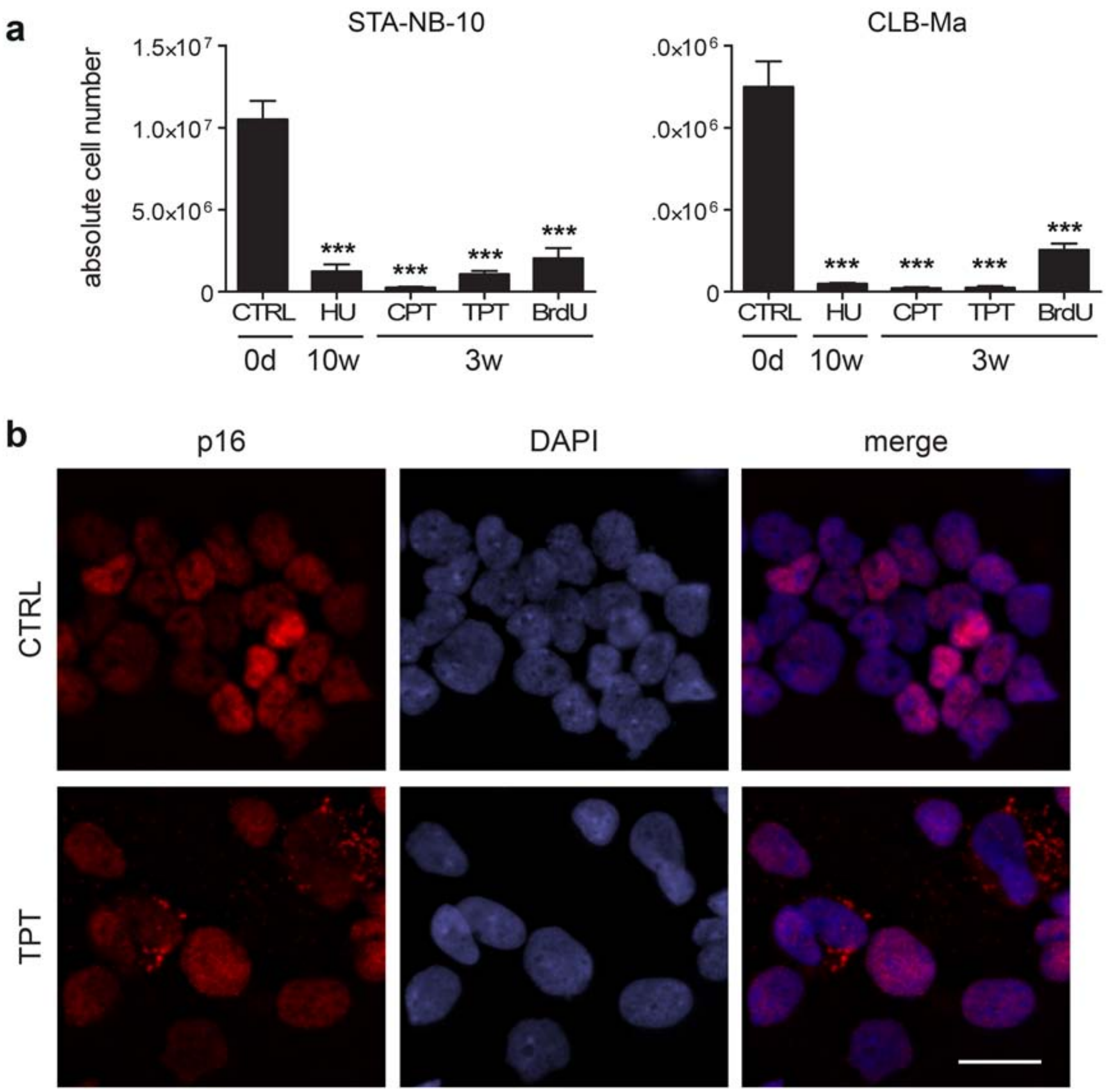

Supplementary Figure S1: Proliferation and gene expression in response to drug-treatment. a. STA-NB-10 (left panel, $n = 4$) or CLB-Ma (right panel, $n = 5$) cell lines treated with the indicated drugs for 3 (CPT, TPT, BrdU) or 10 (HU) weeks, resp., were harvested and counted at the endpoint of treatment. Bar diagrams depict mean absolute cell number/flask +/− SEM at the start (CTRL) or at the treatment endpoint. Asterisks indicate statistically significant differences compared to CTRL. ***$p \leq 0.001$; **$p \leq 0.01$; *$p \leq 0.05$. **b.** p16 IF staining on cytospin preparations of STA-NB-10. bar: 20 µm. Note: focal cytoplasmic IF staining in red is due to increased autofluorescence in TPTsen cells (also visible in green).

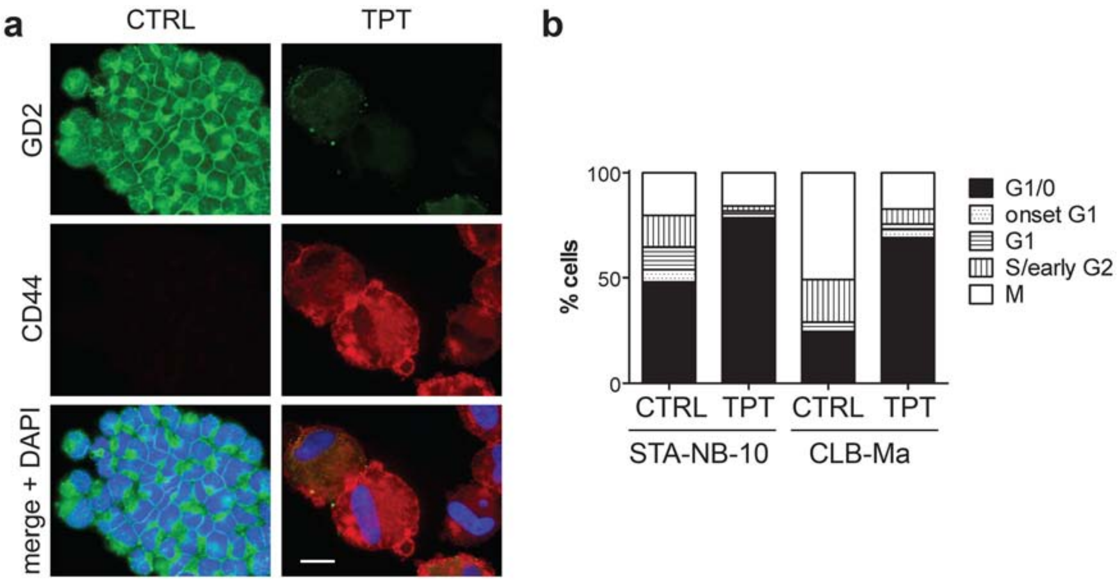

Supplementary Figure S2: Long-term TPT-treated NB-cells are proliferative inactive, display senescence-associated markers, up-regulate CD44 and down-modulate GD2 levels. STA-NB-10 or CLB-Ma cells were cultivated in the absence or presence of 5 nM TPT for 3 weeks. **a.** Analysis of GD2 (green) and CD44 (red) by surface IF staining on cytospin preparations. DAPI has been used as nuclear counterstain (blue).bar: 20 µm **b.** Quantification of Ki-67 staining pattern and corresponding cell cycle state in TPT-treated or untreated control (CTRL) STA-NB-10 and CLB-Ma. bar diagrams depict mean +/− SEM; box plots show mean, box includes 50 percentile, whiskers 10–90 percentile $^{**}p \leq 0.01$; $^{***}p \leq 0.001$;

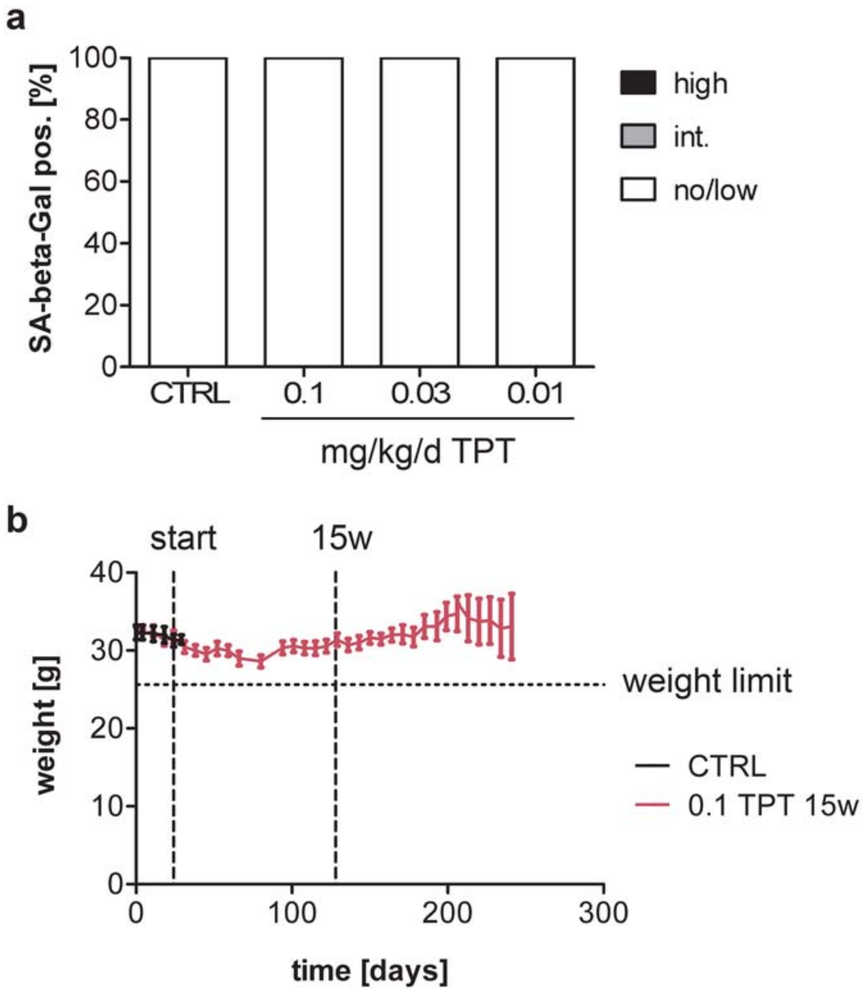

Supplementary Figure S3: a. SA-β-Gal activity in tumor stroma is not affected by low-dose TPT treatment. STA-NB-10 cells were inoculated in CD1nude mice and treatment was started at a mean tumor size of 0.53 +/− 0.2 cm^3 by daily i.p. injection of vector control (CTRL), 1, 0.1, 0.03 or 0.01 mg/kg/d TPT. SA-β-Gal activity of stromal, i.e. mouse, cells located in the tumor area after 2 weeks of treatment. **b.** No significant treatment-related weight loss upon metronomic low-dose topotecan. Curves depict the mean weight/mouse +/− SEM determined 1x/week of mice treated for 15 weeks daily with 0.1 mg/kg/d topotecan (n = 11) or in control animals (n = 5). The dotted line refers to the minimal weight limit as determined by the endpoint criteria (maximal tolerated weight loss of 20% as compared to the initial weight).

SUPPLEMENTARY REFERENCES

1. Ambros IM, Rumpler S, Luegmayr A, Hattinger CM, Strehl S, Kovar H, Gadner H and Ambros PF. Neuroblastoma cells can actively eliminate supernumerary MYCN gene copies by micronucleus formation—sign of tumour cell revertance? European journal of cancer. 1997; 33:2043–2049.
2. Stock C, Bozsaky E, Watzinger F, Poetschger U, Orel L, Lion T, Kowalska A and Ambros PF. Genes proximal and distal to MYCN are highly expressed in human neuroblastoma as visualized by comparative expressed sequence hybridization. The American journal of pathology. 2008; 172:203–214.
3. Combaret V, Turc-Carel C, Thiesse P, Rebillard AC, Frappaz D, Haus O, Philip T and Favrot MC. Sensitive detection of numerical and structural aberrations of chromosome 1 in neuroblastoma by interphase fluorescence in situ hybridization. Comparison with restriction fragment length polymorphism and conventional cytogenetic analyses. International journal of cancer Journal international du cancer. 1995; 61:185–191.
4. Sekiguchi M, Oota T, Sakakibara K, Inui N and Fujii G. Establishment and characterization of a human neuroblastoma cell line in tissue culture. The Japanese journal of experimental medicine. 1979; 49:67–83.
5. Corvi R, Savelyeva L and Schwab M. Duplication of N-MYC at its resident site 2p24 may be a mechanism of activation alternative to amplification in human neuroblastoma cells. Cancer research. 1995; 55:3471–3474.
6. Savelyeva L, Corvi R and Schwab M. Translocation involving 1p and 17q is a recurrent genetic alteration of human neuroblastoma cells. American journal of human genetics. 1994; 55:334–340.
7. Biedler JL, Helson L and Spengler BA. Morphology and growth, tumorigenicity, and cytogenetics of human neuroblastoma cells in continuous culture. Cancer research. 1973; 33:2643–2652.
8. White PS, Maris JM, Beltinger C, Sulman E, Marshall HN, Fujimori M, Kaufman BA, Biegel JA, Allen C, Hilliard C, Valentine MB, Look AT, Enomoto H, Sakiyama S and Brodeur GM. A region of consistent deletion in neuroblastoma maps within human chromosome 1p36.2-36.3. Proceedings of the National Academy of Sciences of the United States of America. 1995; 92:5520–5524.
9. Shapiro DN, Valentine MB, Rowe ST, Sinclair AE, Sublett JE, Roberts WM and Look AT. Detection of N-myc gene amplification by fluorescence in situ hybridization. Diagnostic utility for neuroblastoma. The American journal of pathology. 1993; 142:1339–1346.
10. Vermeulen J, De Preter K, Naranjo A, Vercruysse L, Van Roy N, Hellemans J, Swerts K, Bravo S, Scaruffi P, Tonini GP, De Bernardi B, Noguera R, Piqueras M, Canete A, Castel V, Janoueix-Lerosey I, et al. Predicting outcomes for children with neuroblastoma using a multigene-expression signature: a retrospective SIOPEN/COG/GPOH study. The lancet oncology. 2009; 10:663–671.
11. Vandesompele J, Edsjo A, De Preter K, Axelson H, Speleman F and Pahlman S. ID2 expression in neuroblastoma does not correlate to MYCN levels and lacks prognostic value. Oncogene. 2003; 22:456–460.
